# Prioritization of candidate cancer drugs based on a drug functional similarity network constructed by integrating pathway activities and drug activities

**DOI:** 10.1002/1878-0261.12564

**Published:** 2019-08-21

**Authors:** Jieyi Di, Baotong Zheng, Qingfei Kong, Ying Jiang, Siyao Liu, Yang Yang, Xudong Han, Yuqi Sheng, Yunpeng Zhang, Liang Cheng, Junwei Han

**Affiliations:** ^1^ College of Bioinformatics Science and Technology Harbin Medical University China; ^2^ Department of Neurobiology Harbin Medical University China; ^3^ College of Basic Medical Science Heilongjiang University of Chinese Medicine Harbin China

**Keywords:** drug activities, drug functional similarity network, drug repurposing, pathway activities

## Abstract

Due to the speed, efficiency, relative risk, and lower costs compared to traditional drug discovery, the prioritization of candidate drugs for repurposing against cancers of interest has attracted the attention of experts in recent years. Herein, we present a powerful computational approach, termed prioritization of candidate drugs (PriorCD), for the prioritization of candidate cancer drugs based on a global network propagation algorithm and a drug–drug functional similarity network constructed by integrating pathway activity profiles and drug activity profiles. This provides a new approach to drug repurposing by first considering the drug functional similarities at the pathway level. The performance of PriorCD in drug repurposing was evaluated by using drug datasets of breast cancer and ovarian cancer. Cross‐validation tests on the drugs approved for the treatment of these cancers indicated that our approach can achieve area under receiver‐operating characteristic curve (AUROC) values greater than 0.82. Furthermore, literature searches validated our results, and comparison with other classical gene‐based repurposing methods indicated that our pathway‐level PriorCD is comparatively more effective at prioritizing candidate drugs with similar therapeutic effects. We hope that our study will be of benefit to the field of drug discovery. In order to expand the usage of PriorCD, a freely available R‐based package, PriorCD, has been developed to prioritize candidate anticancer drugs for drug repurposing.

**Abbreviations**
AUROC: area under receiver‐operating characteristic curve
BR: breast
CCLs: cancer cell lines
CMap: Connectivity Map
CNS: central nervous system
CO: colon
FDA: Food and Drug Administration
FDR: false discovery rate
FPR: false‐positive rate
GI50: 50% growth inhibition
KEGG: Kyoto Encyclopedia of Genes and Genomes
LC: lung
LE: leukemia
LOOCV: leave‐one‐out cross‐validation
ME: melanomas
OV: ovary
PCC: Pearson correlation coefficient
PPI: protein–protein interactions
PriorCD: prioritization of candidate drugs
PR: prostate
RE: kidney
RWR: random walk with restart
ssGSEA: single sample gene set enrichment analysis
TPR: true‐positive rate

## 1 Introduction

The research and development of new drugs, especially effective cancer drugs, is a slow and costly process (approximately 12 years and US$1.8 billion on the average) (Sinha and Vohora, 2018). Due to the high attrition rates (most drugs fail due to insufficient safety and/or efficacy) and long time frame for drug development, repurposing drugs (finding new indications for existing drugs) has emerged as an attractive proposition because of lower costs and shorter development times (Ashburn and Thor, 2004; Pushpakom *et al*., 2018). A number of computational methods have been reported to reposition drugs that make use of knowledge in areas such as chemical informatics, bioinformatics, and systems biology to implement the repurposing process based on prior knowledge, broad signatures of activities (e.g., gene expression profiles), or other methods, and each of these has strengths and weaknesses (Jin and Wong, 2014).

Knowledge‐based methods are those using available information to do drug‐repurposing studies, including chemical structure of drugs, adverse effects, protein–protein interactions (PPI), and Food and Drug Administration (FDA) approval labels. Such as PREDICT (Gottlieb *et al*., 2011), this method builds classification features by using known drug–indication associations, as well as drug–drug and indication–indication similarities, and they are subsequently used to predict new drug–indication associations. The advantage of the knowledge‐based methods is that it collects and uses a wealth of prior knowledge, which improves the predictive accuracy of drug repurposing. Although they may have high statistical significance, they involve in fewer molecular‐level mechanisms, such as significantly differential expressed gene compared with signature‐based methods (Jin and Wong, 2014).

There are many signature‐based methods that have been published. The Connectivity Map (CMap) proposed by Lamb *et al*. (2006), a large‐scale algorithm designed to explore functional interactions between drugs as well as between drugs and diseases, is based on the reverse correlations between the drug‐ and disease‐induced gene expression profiles. Another classical method was reported by Shigemizu *et al*. (2012), whose premise rests on using gene expression profiles that significantly changed in normal and cancer cell lines to find candidate drugs that can bring abnormal processes of disease states back to normal (down‐regulate overexpressed genes or up‐regulate underexpressed genes). Such methods generally take the perspective that a drug might have a chance to treat a disease whether there is an inverse correlation between the gene expression profiles after taking drug and that under the disease condition. However, from a system perspective, drugs generally exert the therapeutic effect to the diseases on biological pathways, and both of the methods above were focused on the changes of gene‐level expression. The pathway‐based method may have a potential for drug repurposing and improve the success rate of drug development (Jin and Wong, 2014; Pushpakom *et al*., 2018).

A biological pathway is a series of actions among interacting genes and/or molecules in a cell that leads to a certain product or a change in a cell (Kanehisa and Goto, 2000). Compared to pathway‐based analysis, gene signature‐based analysis often yields a series of genes that are statistically significant but cannot be defined for any single theme on a biological level and thus misses significant impacts on pathways, such as transcriptional regulation and metabolic processes. These cellular processes are generally regulated by several genes acting together, instead of in isolation, and generally do not manifest as changes in individual genes (Subramanian *et al*., 2005; Ye *et al*., 2012). Therefore, understanding the functional similarity of drug effects at the pathway level is helpful to drug repurposing.

Moreover, the National Cancer Institute (NCI)‐60 panel provides data for molecular profiles (e.g., mRNA and microRNA expression profiles) and drug activities for the NCI‐60 cancer cell lines. The drug activities are expressed as the negative log of the concentration that results in a 50% growth inhibition (GI50) in the NCI‐60 cell lines. The NCI‐60 data could be used to study the relationships between expression levels of various mRNA and microRNA, as well as their correlations with drug activity, and these correlations may provide new perspectives for the computational methods of drug repurposing (Shankavaram *et al*., 2009).

Here, we present a novel approach, termed prioritization of candidate drugs (PriorCD), to prioritize candidate cancer drugs by applying a global network propagation algorithm to a drug functional similarity network. We first enriched mRNA and microRNA in the NCI‐60 panel into mRNA and microRNA pathways. Then, the mRNA and microRNA pathway activity profiles were correlated to drug activity profiles to obtain mRNA‐ and microRNA‐based pathway–drug correlations. Subsequently, we measured the correlations among drugs across the pathway activities to construct an mRNA pathway‐based and microRNA pathway‐based drug–drug functional similarity network, which were then integrated into one integrated network. Based on the network, we could make rational biological interpretation on drug functional similarities. Obviously, drugs that are closer and more connected to each other in the functional drug similarity network are more likely to share similar functions and exert similar therapeutic effects on the same disease. After mapping known cancer drugs to the network, we applied a global network propagation algorithm to score candidates by proximity to all known cancer drugs.

In this work, we provide predictions of 14 and 8 candidate drugs for breast cancer and ovarian cancer drug sets, respectively, and compare our results with two other classical drug‐repurposing methods. According to cross‐validation test and receiver‐operating characteristic (ROC) curve analysis, we validated that PriorCD can efficiently prioritize candidate cancer drugs.

## 2 Materials and methods

### 2.1 Data sources and data processing

#### 2.1.1 Chemical compounds anticancer activity data

**Figure 1:**
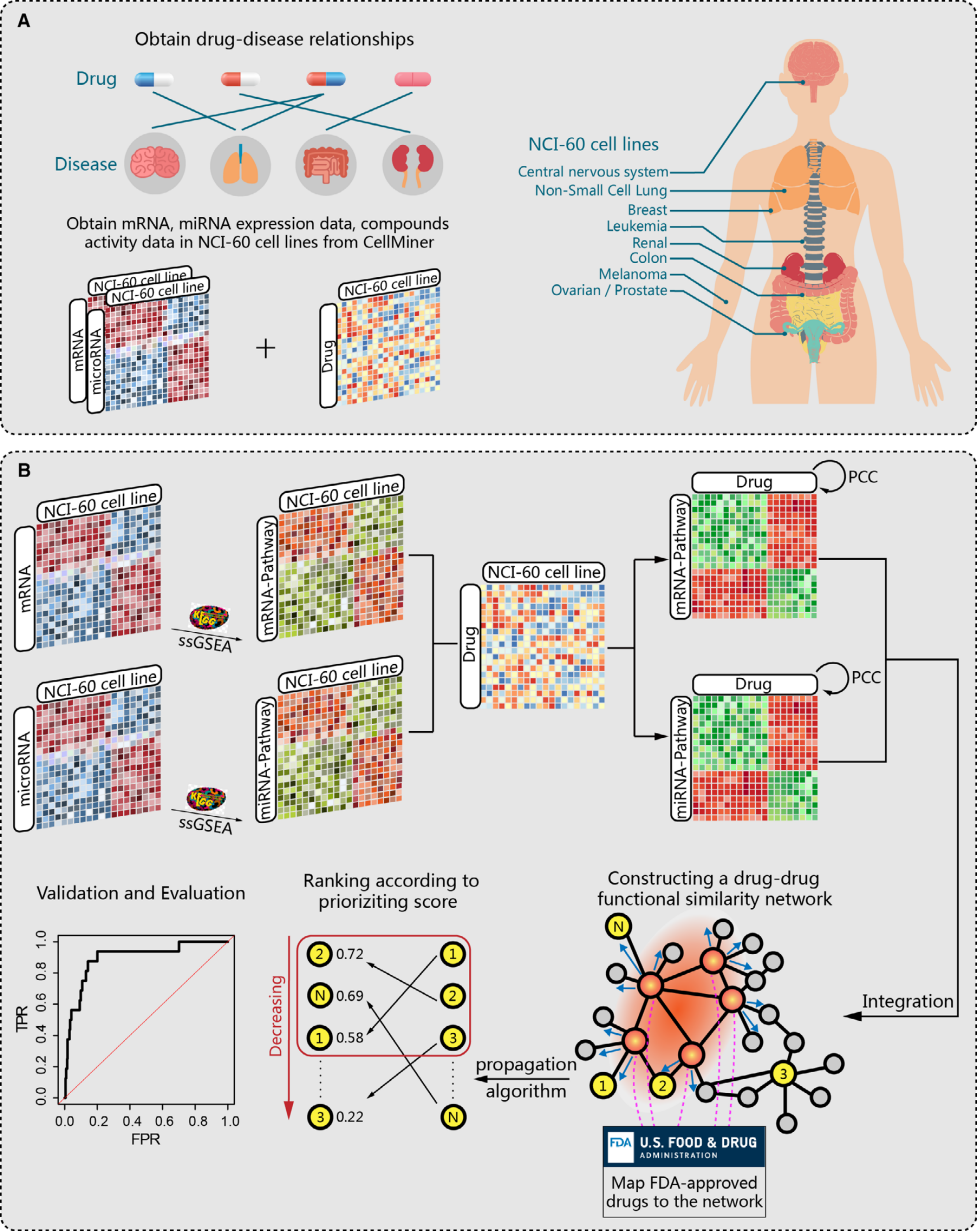
Workflow of PriorCD. (A) Data preparation. Drug–disease relationships were collected from the FDA; mRNA and microRNA expression data and drug activity profiles in NCI‐60 cell lines were obtained from CellMiner. (B) Both mRNA and microRNA expression data were enriched into mRNA and microRNA pathway activity profiles, respectively, and then correlated with drug activity profiles to calculate mRNA‐ and microRNA‐based pathway–drug correlations across NCI‐60 cell lines. Based on these correlations, the functional similarity between each pair of drugs was calculated, and a drug‐drug functional similarity network was then generated. Through mapping of known cancer therapeutic drugs to the network, a global network propagation algorithm was subsequently applied to the network to achieve a prioritized list of drugs, which was validated by ROC curve analysis.

The term ‘drug’ was used to indicate chemical compounds in the study. We collected chemical compounds’ anticancer activity data in NCI‐60 cancer cell lines (CCLs) from the CellMiner database (Shankavaram *et al*., 2009), which comprises up to 20 000 compounds. The NCI‐60, a panel of 60 human CCLs from nine tissues of origin widely used in the study of drug discovery and cancer biology to screen chemical compounds for anticancer activity (Blower *et al*., 2007), includes melanomas (ME), leukemia (LE), and cancers of the breast (BR), lung (LC), ovary (OV), prostate (PR), central nervous system (CNS), kidney (RE), and colon (CO) (Fig. 1A). The activity levels are expressed as the negative log of the half maximal growth inhibition concentration [−log10(GI50)], which denotes that higher values equate to higher sensitivity of cell lines. For drugs with duplicate IDs, we aggregated their activity data by means. To limit our drug set, which showed relatively high and diverse activity across NCI‐60 CCLs, we calculated two values, the inter‐quartile range (IQR) and maximum intensity for each drug. In the end, 3645 drugs were retained for our analyses that were contained in both the top quartile of the IQR and the top quartile of maximum intensity.

#### 2.1.2 mRNA and microRNA expression data

In order to better analyze and understand the effects of drugs on multiple levels, we collected mRNA and microRNA expression data for the NCI‐60 CCLs separately; 19 794 mRNA expression data in NCI‐60 CCLs normalized by GC robust multi‐array average (GCRMA) from Affymetrix Human Genome U133 Plus 2.0 microarrays (Reinhold *et al*., 2010) and 319 microRNA expression data in NCI‐60 CCLs normalized by log2 from OSU V3 microarray (Blower *et al*., 2007; Gaur *et al*., 2007) were retrieved from CellMiner (Fig. 1A). For mRNA and microRNA with duplicate IDs, we aggregated their expression values by means for our analysis.

### 2.2 Workflow overview

PriorCD was developed to prioritize candidate compounds against a cancer of interest for drug repurposing based on a drug functional similarity network. Our method consists of four main steps: (a) inferring mRNA and microRNA pathway activity profiles, (b) constructing a functional similarity network between drugs by integrating mRNA and microRNA pathway‐based drug similarities, (c) calculating drug prioritizing scores according to a set of approved therapeutic drugs for the cancer of interest based on a global network propagation algorithm, and (d) evaluating the statistical significance of drug prioritized scores by random permutation test and measuring the performance of the prioritizing procedure by leave‐one‐out cross‐validation (LOOCV). A flow diagram of the PriorCD methodology is shown in Fig. 1. PriorCD has been implemented as a freely available R‐based tool (https://cran.r-project.org/web/packages/PriorCD). Users need to input a set of approved therapeutic drugs for a particular cancer, and then, the prioritized list of candidate drugs will be returned.

### 2.3 Inferring mRNA and microRNA pathway activity profiles

Single sample gene set enrichment analysis (ssGSEA) (Barbie *et al*., 2009) against 250 curated gene sets (C2) of Kyoto Encyclopedia of Genes and Genomes (KEGG) pathways from MSigDB (http://software.broadinstitute.org/gsea/msigdb/index.jsp) was carried out to convert mRNA expression data into mRNA pathway activity profiles; then, 227 resultant mRNA pathways were extracted. For microRNA expression data, we first obtained microRNA–mRNA interaction data from our previous study (Han *et al*., 2016), where we converted pathways of mRNA into pathways of microRNA. Then, the target mRNA of microRNA were mapped into mRNA pathways and a hypergeometric test was used to calculate the *P*‐values of the associations between microRNA and mRNA pathways. The associations with *P*‐value < 0.05 were considered to be microRNA pathways, which were used in our work to obtain microRNA pathway activity profiles. In total, 124 microRNA pathways had been enriched. These pathway activity profiles were then used to obtain pathway–drug correlations and subsequently pathway‐based drug correlations.

### 2.4 Constructing a drug–drug functional similarity network

Our method for drug repurposing is based on the observation that drugs in the vicinity of the approved therapeutic drugs within the network are more likely to share similar biological effects. We sought to reveal the similarity of anticancer activity between drugs on the biological pathway level. Toward this goal, we first performed a Pearson correlation analysis between mRNA pathway or microRNA pathway activity profiles and drug activity data across all NCI‐60 CCLs. The Pearson correlation coefficient (PCC) reflects the extent of correlation between pathways and drugs.

Next, based on the resultant pathway–drug correlation matrices, individual relationships between drugs on the mRNA and microRNA pathway levels were defined based on the PCC between each pair of drugs, which describes the functional similarity between activity patterns of the drug pairs across all of the pathways. The resulting *P*‐values of PCC were false discovery rate (FDR) adjusted to correct for multiple comparisons. We then constructed an integrated drug functional similarity network whose vertices were drugs and edges represented significant functional similarity as follows: For each drug, we considered drugs with correlation coefficient ≥ 0.7, FDR ≤ 0.05, and drugs that ranked in top 0.05% of decreasing correlation coefficients as significantly similar. Drug functional similarity networks based on mRNA and microRNA pathways were then merged. Vertices that had edges in any of the networks constructed above were also connected in the integrated network. Approximately 82 000 undirected edges among 3645 drugs were contained in this network. This drug functional similarity network is provided in our package and can also be downloaded from the Table S1. Drugs with similar activity patterns under multiple but similar experimental conditions have higher probability of being involved in related biological pathways and treating similar diseases. Using an integrated drug similarity network, it is feasible to capture the subtle functional relationships among drugs. This integrated drug similarity network can be represented as a drug–drug functional similarity matrix, which can be used in the following analysis.

### 2.5 Calculating drug prioritizing scores

Our drug‐repurposing process exploits random walk with restart algorithm (RWR) (Kohler *et al*., 2008) on the integrated drug similarity network. RWR is a global network propagation algorithm for quantifying similarity between any given node of a network and a given set of nodes called the restart set, because the complete network structure is traversed during these iterations. In a random walk, a set of start nodes (restart set) in the network is defined, here corresponding to approved therapeutic drugs against a specific cancer. In each iteration, the random paths are extended from their current nodes, and either transition to a neighboring network node or jump to one of the nodes in the restart set with a certain restart probability. Each node in the network is assigned a probability describing the chance of visiting. When reaching the steady state, nodes in the network are ranked by their visiting probabilities (Fig. 1B). The visiting probability of each node determines the similarity between the restart set and that node. Those with high visiting probability are more proximal and more similar to the restart set and more likely to be considered as candidates. This algorithm has prioritized disease genes in many other studies (Kohler *et al*., 2008).

As input, RWR accepts a set of approved therapeutic drugs for a cancer of interest, an undirected drug similarity network, and a restart probability. The random walk with start process is described as Eqn (1):

(1) $$p^{t}=\left(1-\alpha \right)Ap^{t-1}+\alpha p^{0}$$

where *p* ^1 ^= *p* ^0^; *p* ^*t*^ is a vector containing visiting probabilities of all nodes in the network at time point *t*. *A* is a column‐normalized adjacent matrix of the drug similarity network. *p* ^0^ represents the initial probability vector of nodes, where the nodes in the restart set corresponding to approve therapeutic drugs against a specific cancer are assigned as 1 and remaining nodes as 0. These binary numbers represent the prior knowledge of the drugs. The factor *α* ∈ (0,1) is a certain probability of continuing the random walk or restarting from the restart set. In this study, *α* was set to be 0.7, because Kohler *et al*. (2008) reported that *α* had only a slight effect on the results of the RWR algorithm when it fluctuated between 0.1 and 0.9. The probability vector *p* ^*t*^ will reach a steady state at certain time point, when the difference between *p* ^*t*^ and *p* ^*t*−1^ falls below 10^−10^, and then, the RWR algorithm will terminate. Drugs were then ranked according to the values in the steady‐state probability vector *p* ^*t*^, which were used as drug prioritizing scores.

### 2.6 Statistical significance analysis and method evaluation

To stringently compare with randomized networks to access the statistical significance of drug prioritizing scores, we generated degree‐preserving random networks with precisely the same number of edges for each node as in the real drug similarity network but with different node labels. Simultaneously, the adjacency matrices of random networks had the same number of nonzero values in each row and column as the real network. For each random network, we recalculated the prioritized scores of each drug. The *P*‐value of each drug's prioritized score was computed as the ratio of the counts with larger score in the random networks divided by the permutation times. In this study, permutation times were set at 1000 as the default. The FDR was accessed by the Benjamin–Hochberg method (Benjamini and Hochberg, 1995).

Furthermore, the LOOCV test was applied here to test the performance of our method. For an arbitrary set of approved therapeutic drugs against a specific cancer, in order to perform the LOOCV test, each drug was chosen, and its cancer annotation was then hidden, one at a time within the set. We evaluated the performance of our method by its success rate of re‐annotating the cancer annotation‐removed drugs. Moreover, the ROC curve, which plots the true‐positive rate (TPR) versus the false‐positive rate (FPR), which is subject to the drug prioritizing scores separating the identification results, and the AUROC was then computed to measure the performance of our method.

## 3 Results

### 3.1 Reliability analysis of mRNA and microRNA pathway activity data

In this study, 227 mRNA pathways and 124 microRNA pathways were extracted from mRNA and microRNA expression data in NCI‐60 CCLs by using the ssGSEA method (see Section 2).

**Figure 2:**
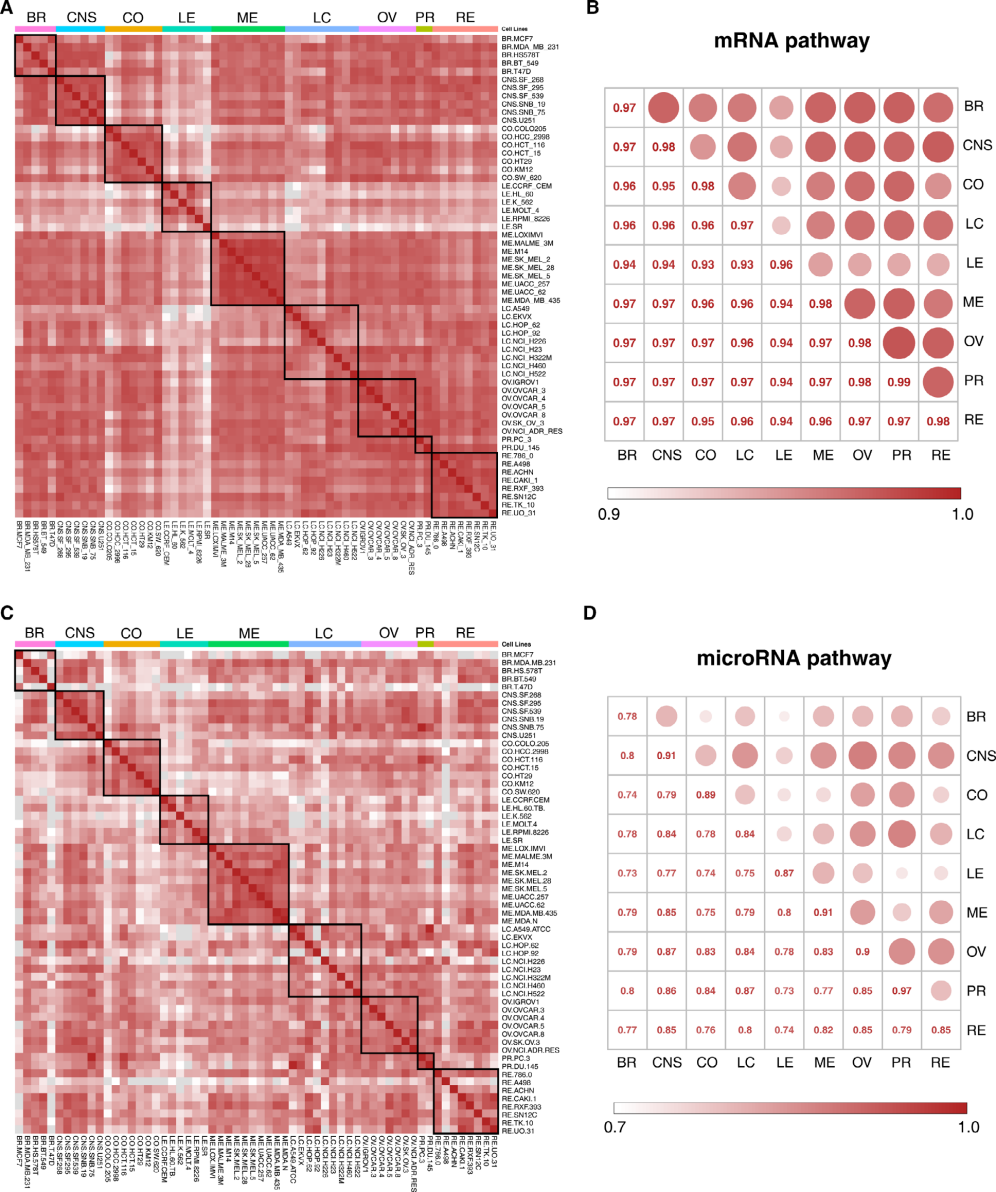
Cell–cell and tissue‐of‐origin correlation. Pearson correlation coefficient (PCC) of 227 mRNA and 124 microRNA pathway activity profiles, respectively, presented at the levels of NCI‐60 cell line and tissue of origin. (A) Heatmap of cell–cell correlation coefficient for mRNA pathway. (B) Mean tissue of origin correlation coefficient for mRNA pathway. (C) Heatmap of cell–cell correlation coefficient for microRNA pathway. (D) Mean tissue of origin correlation coefficient for microRNA pathway.

To assess the technical reproducibility and variation among disparate cell lines for mRNA and microRNA pathway activity data, in which we carried out Pearson correlation analysis for designated cell–cell groups in Fig. 2A,C, respectively, all of the enriched mRNA and microRNA pathways were included. For mRNA pathway activity correlation from different cell lines, the average correlation was 0.960, with a range of 0.869–0.994 and a standard deviation of 0.018 (Fig. 2A). For microRNA pathway activity correlation from different cell lines, the average correlation was 0.806, with a range of 0.465–0.996 and a standard deviation of 0.081 (Fig. 2C).

The tissue‐of‐origin correlations of mRNA pathway activities and those of microRNA pathway activities in Fig. 2B,D, respectively, were calculated using the same pathway activity data as described above, indicating the variation levels both within and between tissues of origin. Averages were taken when a particular tissue of origin comprised multiple cell lines. For mRNA pathway activity correlation within a single tissue of origin, the average correlation was 0.977, with a range of 0.964–0.987 and a standard deviation of 0.008. For that between different tissues of origin, the average correlation decreased to 0.959 with a range of 0.926–0.977 and a standard deviation of 0.014 (Fig. 2B). For microRNA pathway activity correlation within a single tissue of origin, the average correlation was 0.880, with a range of 0.777–0.965 and a standard deviation of 0.053. For that from different tissues of origin, the average correlation dropped to 0.799, with a range of 0.726–0.874 and a standard deviation of 0.042 (Fig. 2D).

We could observe in Fig. 2B,D that microRNA pathway activity profile has greater variation than the mRNA pathway activity data. In addition, melanoma (ME) and central nervous system (CNS) were the most coherent (their correlation was 0.9831 and 0.9833, respectively), whereas leukemia (LE) and lung cancer (LC) were the least coherent (their correlation was 0.9638 and 0.9667 respectively). In order to explore which pathways are responsible for these correlations or distinctions, we selected six leukemia (LE) cell lines and nine lung cancer (LC) cell lines as an example. Figure S1 shows the hierarchical clustering heatmap of 15 cancer cell lines and 227 mRNA pathways. We could clearly observe that cancer cell lines from the same tissue are clustered together. In addition, many pathways had almost the same activity pattern, that is, consistently high activity or low activity. Such as the activity of citrate cycle (TCA cycle) pathway was consistently high, but the activity of olfactory transduction pathway was consistently low. Also, some pathways acted distinctly different in the two tissues. For example, ECM‐receptor interaction pathway and histidine metabolism pathway had lower activity in leukemia cell lines and higher activity in lung cancer cell lines. On the contrary, carbohydrate digestion and absorption pathway had higher activity in leukemia cell lines and lower activity in lung cancer cell lines. Therefore, the pathways having the same activity pattern in different tissues may lead to high correlations and pathways having reverse activity pattern in different tissues may be responsible for the distinction of tissues.

In comparison with the cell–cell and tissue‐of‐origin correlations based on pathway activity levels (Fig. 2), the correlations based on gene expression levels are lower (Fig. S2). For instance, for mRNA expression correlation from different cell lines, the average correlation was 0.886, with a range of 0.810–0.981 and a stand deviation of 0.027. Within a single tissue of origin, the average mRNA expression correlation was 0.930, with a range of 0.908–0.952 and a stand deviation of 0.016. For that between different tissues of origin, the average correlation was 0.883, with a range of 0.840–0.901 and a stand deviation of 0.020.

As shown in Fig. 2, some unrelated cell lines and tissues of origin seemingly had high correlations, which are likely generated by KEGG pathways representing housekeeping processes and those representing processes that are not expressed in cancer cell lines. Moreover, the length of pathway activity profile (*n* = 227) is much shorter than the length of gene expression profile (*n* > 10 000), which could also lead to high correlations.

These results indicated that in comparison with using mRNA and microRNA expression data, using mRNA and microRNA pathway activity data demonstrated the coherence of pathway activities within tissues as well. Moreover, pathway activity profiles contain fewer features and smaller fluctuation. Therefore, it is more appropriate for us to use pathway activity profiles to analyze functional similarities among drugs.

**Figure 3:**
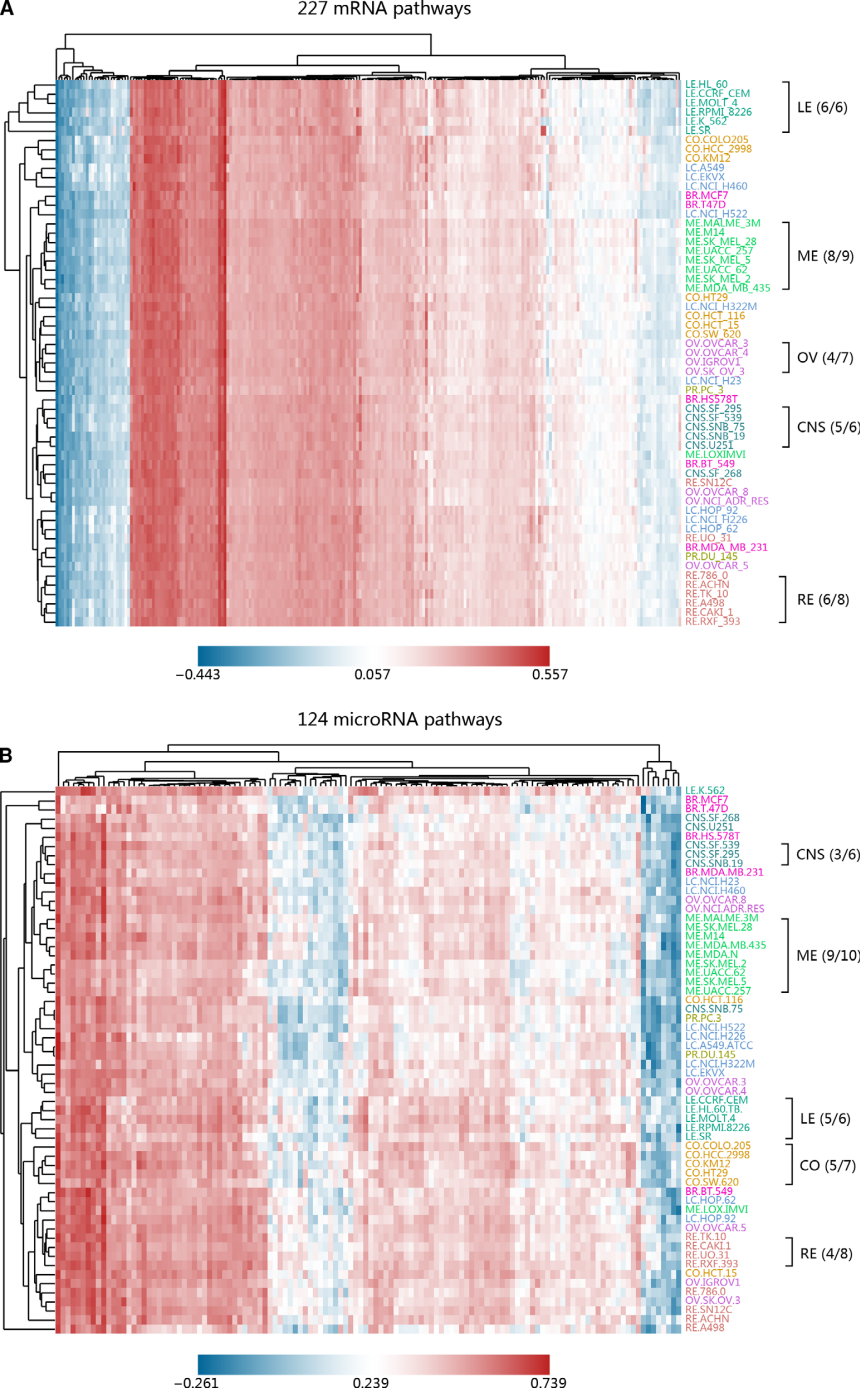
Clustered image of (A) 227 mRNA and (B) 124 microRNA pathway activity levels in NCI‐60 cell lines, where red indicates high activity level and blue indicates low activity level.

The clustering results of 227 mRNA pathways and 124 microRNA pathways are shown in Fig. 3A,B, where high activity levels are expressed in red and low activity levels in blue. Separation according to tissues of origin for both mRNA and microRNA pathway activity data was observed in most instances when cell lines were clustered. Particularly noteworthy is that cell lines from the same tissue of origin tended to be clustered together. For mRNA pathway activity data, six leukemia cell lines (six in total) and eight melanoma cell lines (nine in total) were clustered together (Fig. 3A). For microRNA pathway activity data, five leukemia cell lines (six in total) and nine melanoma cell lines (10 in total) were clustered together (Fig. 3B), which indicated relatively high coherence between mRNA and microRNA pathway activity.

Since the results of using mRNA and microRNA pathways were consistent with, or even better than, the results of using mRNA and microRNA in Liu *et al*., it was theoretically feasible to analyze drug effects on the pathway level. Furthermore, using pathway activity data enables more comprehensive and accurate results, which was more conducive to capturing, researching and summarizing the similarity of drug effects from the biological functional level (Liu *et al*., 2010).

### 3.2 Prioritization of drugs for breast cancer

Breast cancer, the second cause of death worldwide among females in recent years (Ferlay *et al*., 2015), is also a very important model to evaluate new therapeutic drugs in cancer research. Therefore, we collected 33 FDA‐approved drugs for treating breast cancer from the NCI (National Cancer Institute) at the NIH (National Institutes of Health) website (https://www.cancer.gov/about-cancer/treatment/drugs/breast) on January 2, 2019. Biologics drugs, such as proteins, antibodies, cell therapies, and lytic viruses, were excluded because they did not consist in our chemical compound datasets and did not make sense based on our fundamental approach. After excluding these, 16 breast cancer drugs remained in this section for analysis, which was concerned as the restart set and summarized in Table S2.

Drugs such as paclitaxel (antimitotic agent), lapatinib ditosylate (EGFR and ErbB‐2 dual tyrosine kinase inhibitor), and fluorouracil (DNA and RNA synthesis inhibitor) are FDA‐approved drugs for treating breast cancer that are included in our restart set, which were first mapped into our drug functional similarity network. The RWR algorithm was then performed on our network to prioritize drugs. With FDR < 0.001, PriorCD identified 14 statistically significant candidate drugs, which may potentially treat breast cancer (Table 1). The full list of ranked drugs is listed in the Table S3.

**Table 1:** Candidate drugs for breast cancer identified by PriorCD with FDR < 0.001

NSCID	Drug name	Prior score	FDR	Status^a^	M.O.A.^b^
715055	Gefitinib	9.73E‐03	< 0.001	FDA approved	YK|PK:EGFR
750691	Afatinib	3.14E‐03	< 0.001	FDA approved	YK|PK:EGFR
761910	Ibrutinib	3.14E‐03	< 0.001	FDA approved	YK
693255	Tyrphostin AG 1478	3.13E‐03	< 0.001	–	YK
677423	Amythiamicin a	1.69E‐03	< 0.001	–	–
673191	–	1.58E‐03	< 0.001	–	–
668404	–	1.39E‐03	< 0.001	–	–
123139	l‐cysteine, s‐[(4‐methylphenyl)diphenylmethyl]‐(9ci)	1.38E‐03	< 0.001	–	–
164011	Zorubicin	1.15E‐03	< 0.001	–	–
82151	Daunorubicin	1.13E‐03	< 0.001	FDA approved	T2
711946	Antineoplastic‐d668094	1.10E‐03	< 0.001	–	–
736681	–	1.07E‐03	< 0.001	–	–
726148	n,n’‐bis[4‐(n‐butylamidino)phenyl}homopiperazine	1.05E‐03	< 0.001	–	–
699491	Epidoxoform	7.74E‐04	< 0.001	–	–

a Status is the current stage of drugs, which can be divided into FDA approved, Europe approved, clinical trial, and none (–).

b M.O.A. is the abbreviation of mechanism of action, and detailed information can be found in the Table S9.

Specially, there are 14 prioritized candidate drugs in total (Table 1). Gefitinib (prioritized score = 9.73E‐03, FDR < 0.001), afatinib (prioritized score = 3.14E‐03, FDR < 0.001), ibrutinib (prioritized score = 3.14E‐03, FDR < 0.001), tyrphostin AG1478 (prioritized score = 3.13E‐03, FDR < 0.001), zorubicin (prioritized score = 1.15E‐03, FDR < 0.001), and daunorubicin (prioritized score = 1.13E‐03, FDR < 0.001) show significant prioritized scores in our PriorCD method and are considered to show great potential therapeutic effects in the treatment of breast cancer.

Gefitinib (NSC715055), a type of epidermal growth factor receptor (EGFR) tyrosine kinase inhibitor, is an FDA‐approved drug for treating non‐small‐cell lung cancer (NSCLC). EGFR is an established therapeutic target in the treatment of breast cancer. The over‐expression of EGFR in breast cancer is associated with poor differentiation and prognosis (Masuda *et al*., 2012; Rimawi *et al*., 2010). Kalykaki *et al*. (2014) showed that gefitinib had encouraging clinical benefits (clinical trial: NCT00428896) in eliminating circulating tumor cells in metastatic breast cancer.

Afatinib (NSC750691) is an orally administered blocker of the tyrosine kinase and epidermal growth factor receptor family, with antineoplastic activity. It has been approved by FDA for the first‐line treatment of NSCLC. The positive therapeutic effect of afatinib in the treatment of breast cancer, in particular trastuzumab‐resistant HER2‐positive breast cancer, has been observed in phase I/II clinical studies (Canonici *et al*., 2016; Hurvitz *et al*., 2014; Lin *et al*., 2012). Additionally, the study of its effect in combination with letrozole suggested a potential in advanced hormone‐refractory breast cancer (Gunzer *et al*., 2016).

Ibrutinib (NSC761910), an orally bioavailable small molecular drug, can bind irreversibly to inhibit Bruton's tyrosine kinase (BTK) activity. It has been approved and shows notable clinical antineoplastic activity against several B‐cell lymphoproliferative diseases, such as chronic lymphocytic leukemia (CLL), small lymphocytic lymphoma (SLL), and Waldenström macroglobulinemia (a type of non‐Hodgkin lymphoma). Ibrutinib has been reported to play a valuable role in inhibiting activity of BTK‐C, a novel isoform of BTK that protects breast cancer cells from apoptosis (Wang *et al*., 2016). Specifically, the effect of ibrutinib has been clearly confirmed in the suppression of the growth of HER2+ breast cancer cell lines (Chen *et al*., 2016). Thus, it could become a drug for the treatment of ErbB2+ breast cancer (Campbell *et al*., 2018; Grabinski and Ewald, 2014).

Tyrphostin AG1478 (NSC693255), a potent and specific quinazoline small molecular inhibitor of EGFR tyrosine kinase (Lenferink *et al*., 2000; Zhang *et al*., 2008), is another compound we consider to be a prioritized candidate in treatment of breast cancer. An *in vitro* study showed that the cytotoxicity of EGFR inhibitor tyrphostin AG1478 on breast cancer cell lines was enhanced when simultaneously suppressing the phosphoinositide 3‐kinase (PI3K) signaling pathway, aberrant activation and dysfunction of which were frequently reported in breast carcinogenesis (Li *et al*., 2012).

Daunorubicin (NSC82151), an anthracycline chemotherapeutic, inhibits the replication and repair of DNA and the synthesis of RNA and protein, and was approved by the FDA to treat acute leukemia, that is, acute lymphoblastic leukemia (ALL) and acute myeloid leukemia (AML). The valuable anticancer activity of stealth liposomal daunorubicin in eliminating breast cancer cell has been validated in *in vitro* studies by Guo *et al*. (2010). Moreover, octreotide‐modified daunorubicin liposomes could potentially prevent breast cancer invasion according to Ju *et al*. (2018) and Liu *et al*. (2017). In addition, zorubicin (NSC164011), a benzoylhydrazine analog of daunorubicin, which shares similar effects with daunorubicin in inhibition of carcinogenesis, is now in a phase III clinical trial for treatment of breast cancer (Jeswani and Paul, 2017). Meanwhile, the reason why daunorubicin (NSC82151) and zorubicin (NSC164011) could be regarded as candidate drugs can also be found in our drug functional similarity network. A subnet of the drug functional similarity network was extracted (Fig. S3), displaying the network structure of part of three drugs in restart set (red nodes) and five candidate drugs (yellow nodes). The prioritized scores of each drug were determined by the global distance between itself and drugs in the restart set. When they had more direct neighbors and more shared neighbors (i.e., indirect connections via hub nodes, gray nodes) with the restart set, the RWR algorithm was more inclined to propagate to these drugs and give them higher prioritized score. This indicates that the candidate drugs would possess greater functional similarity with the drugs in the restart set. For instance, daunorubicin and zorubicin have more counts of direct (3, 3) and shared (39, 34) neighbors in drugs in the restart set than other drugs in the subnet, thus their prioritized scores are higher (Fig. S3).

### 3.3 Prioritization of drugs for ovarian cancer

Ovarian cancer has a high incidence worldwide. It is estimated that there are about 239 000 new cases and 125 000 fatalities per year worldwide, which is also the eighth leading cause of death in females (Ferlay *et al*., 2015). Furthermore, the 5‐year relative survival rate of ovarian cancer at the last stage is only 1 in 29 (Reid *et al*., 2017). Therefore, in order to enhance the cure rates and to weaken the toxicity of side effect of current treatment regimens for ovarian cancer, the discovery of treatments for ovarian cancer is imperative. At present, the FDA has approved more than 10 drugs for the treatment of ovarian cancer. We visited NIH website (https://www.cancer.gov/about-cancer/treatment/drugs/ovarian) on January 2, 2019, to collect these drugs. After cross‐referencing with our drug list, seven drugs were left in the case study of ovarian cancer and considered as a restart set and is summarized in Table S2.

Drugs that are used to treat ovarian cancer, for instance, gemcitabine (antimetabolite with antineoplastic activity), doxorubicin (anthracycline antibiotic with activity of topoisomerase II inhibition), and topotecan (camptothecin derivative, inhibitor of topoisomerase I), are approved by the FDA for the treatment of different types of ovarian cancer widespread and used as a restart set in our method. The procedure of prioritization was done as before.

For a new treatment plan for ovarian cancer, eight drugs are identified by PriorCD as candidate drugs, which may become effective strategies of the treatment for ovarian cancer. We regard camptothecin and its derivatives, irinotecan and its biologically active metabolite SN38 (prioritized score see Table 2, FDR < 0.001) and epirubicin (prioritized score = 1.66E‐03, FDR < 0.001) as optimal therapeutic candidates. Table 2 comprises detailed information about eight candidate drugs. The full list of ranked drugs is listed in the Table S4.

**Table 2:** Candidate drugs for ovarian cancer identified by PriorCD with FDR < 0.001

NSCID	Drug name	Prior score	FDR	Status^a^	M.O.A.^b^
681644	Camptothecin Derivative	2.03E‐03	< 0.001	–	T1
629971	Camptothecin Derivative	2.03E‐03	< 0.001	–	T1
94600	Camptothecin	1.99E‐03	< 0.001	–	T1
728073	Irinotecan	1.90E‐03	< 0.001	FDA approved	T1
673596	7‐Ethyl‐10‐hydroxycamptothecin	1.86E‐03	< 0.001	FDA approved	T1
711946	Antineoplastic‐d668094	1.74E‐03	< 0.001	–	–
256942	Epirubicin	1.66E‐03	< 0.001	FDA approved	T2
610457	Camptothecin Derivative	1.52E‐03	< 0.001	–	T1

a Status is the current stage of drugs, which can be divided into FDA approved, Europe approved, clinical trial, and none (–).

b M.O.A. is the abbreviation of mechanism of action, and detailed information can be found in the Table S9.

**Figure 4:**
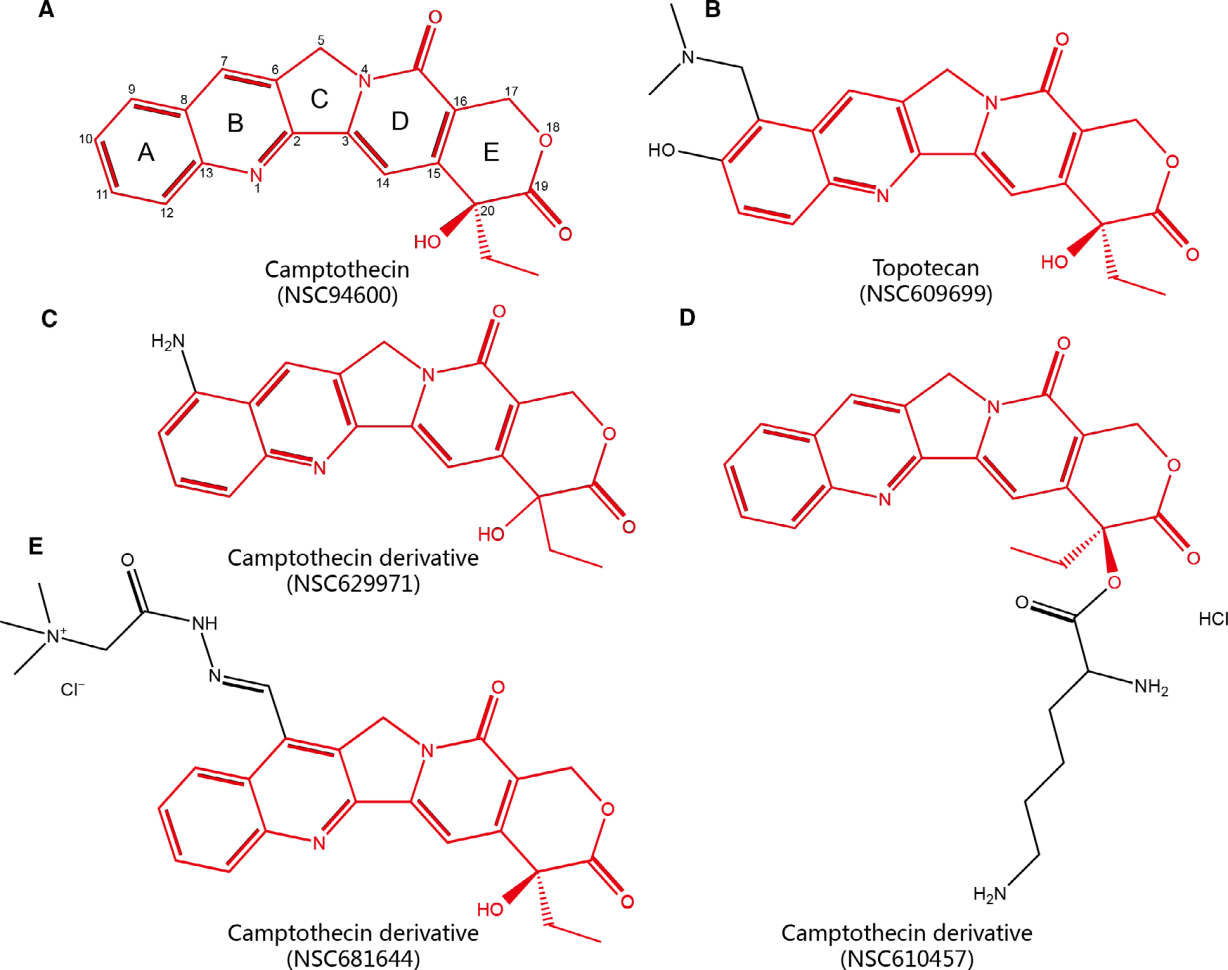
Chemical structures of camptothecin and its derivatives. (A) Camptothecin (NSC94600). (B) Topotecan (NSC609699), FDA‐approved drug for ovarian cancer. (C) Camptothecin derivative NSC629971. (D) Camptothecin derivative NSC610457. (E) Camptothecin derivative NSC681644. Structures in red represent their common structure.

NSC94600, known as camptothecin (Fig. 4A), is a natural quinoline alkaloid isolated from the Chinese tree *Camptotheca acuminata* (Wall *et al*., 1966). It has broad‐spectrum anticancer activity *in vitro*, especially against many solid tumors. Plenty of camptothecin analogs have been synthesized to date, such as irinotecan (for colorectal cancer) and topotecan (for cervical cancer, ovarian cancer, and small cell lung cancer), which have been approved by the FDA for use in the treatment of cancer (Sooryakumar *et al*., 2011). Camptothecin has been reported to exhibit significant preclinical antineoplastic activity in ovarian cancer cell lines (Beggiolin, 2005; Sriram *et al*., 2005). Different types of camptothecin derivatives appeared most frequently in our prioritized list, such as NSC629971 (Fig. 4C), NSC610457 (Fig. 4D), and NSC681644 (Fig. 4E). Despite there not being enough preclinical or clinical trials yet to demonstrate their efficacies, we analyzed them from their chemical structure and found that they have exactly the same parent ring system (Fig. 4A), the pyranoindolizinoquinoline. The substituents on the ring system are the only difference among them. In addition, they are very similar in structure to the FDA‐approved camptothecin derivative topotecan (NSC609699) (Fig. 4B) according to the maximum common substructure (MCS) Tanimoto similarities (0.62, 0.81, and 0.63, respectively, calculating by R‐based package ‘ChemmineR’) (Cao *et al*., 2008). Based on previous studies on the structure–activity relationship (SAR) of camptothecin, the addition of substituents at positions 7, 9, 10, and 11 of the A and B rings can retain or improve its antitumor activity (Li *et al*., 2017; Venditto and Simanek, 2010). Two of three camptothecin derivatives we prioritized have one substituent at the above positions. This also structurally verifies that these camptothecin derivatives may have good effects on preclinical studies and are therefore considered as potential therapeutic drugs for treating ovarian cancer.

Irinotecan (NSC728073), also a camptothecin derivative, is a type of topoisomerase inhibitor approved by the FDA to treat colon or rectal cancer alone or combined with other drugs. It can be converted by carboxylesterase converting enzyme to the active metabolite 7‐Ethyl‐10‐hydroxycamptothecin (SN38, NSC673596) in the body, which is up to 1000 times more active than its prodrug irinotecan. Recent experiments *in vitro* and *in vivo* show that irinotecan has moderate single‐agent activity in treating platinum‐sensitive and platinum‐resistant ovarian cancer (Bodurka *et al*., 2003; Muggia *et al*., 2013). As for attaching cytotoxic drugs to monoclonal antibodies, that is, antibody–drug conjugates (ADCs) of irinotecan attached to bevacizumab, results of clinical phase II studies also show that they have great potential for recurrent ovarian cancer (Muggia *et al*., 2013; Musa *et al*., 2017). In addition, according to Yao *et al*. (2015) trastuzumab‐SN38 conjugates may have encouraging activity in HER2‐positive ovarian cancer.

The topoisomerase II inhibitor epirubicin, a 4′‐epi‐isomer of the anthracycline antibiotic doxorubicin (epirubicin, NSC256942), is also ranked highly in our prioritized list of ovarian cancer drugs. It has been considered to be safe and effective as the first‐line drug in the treatment of metastatic breast cancer through clinical trials (Conte *et al*., 2000). Sayal *et al*. (2015) reported the combination of epirubicin and gemcitabine in the treatment of platinum‐resistant epithelial ovarian cancer (EOC) and provided a new option of ovarian carcinoma treatment, which is likely to become an effective regimen after further investigation.

### 3.4 Performance of the PriorCD method

**Figure 5:**
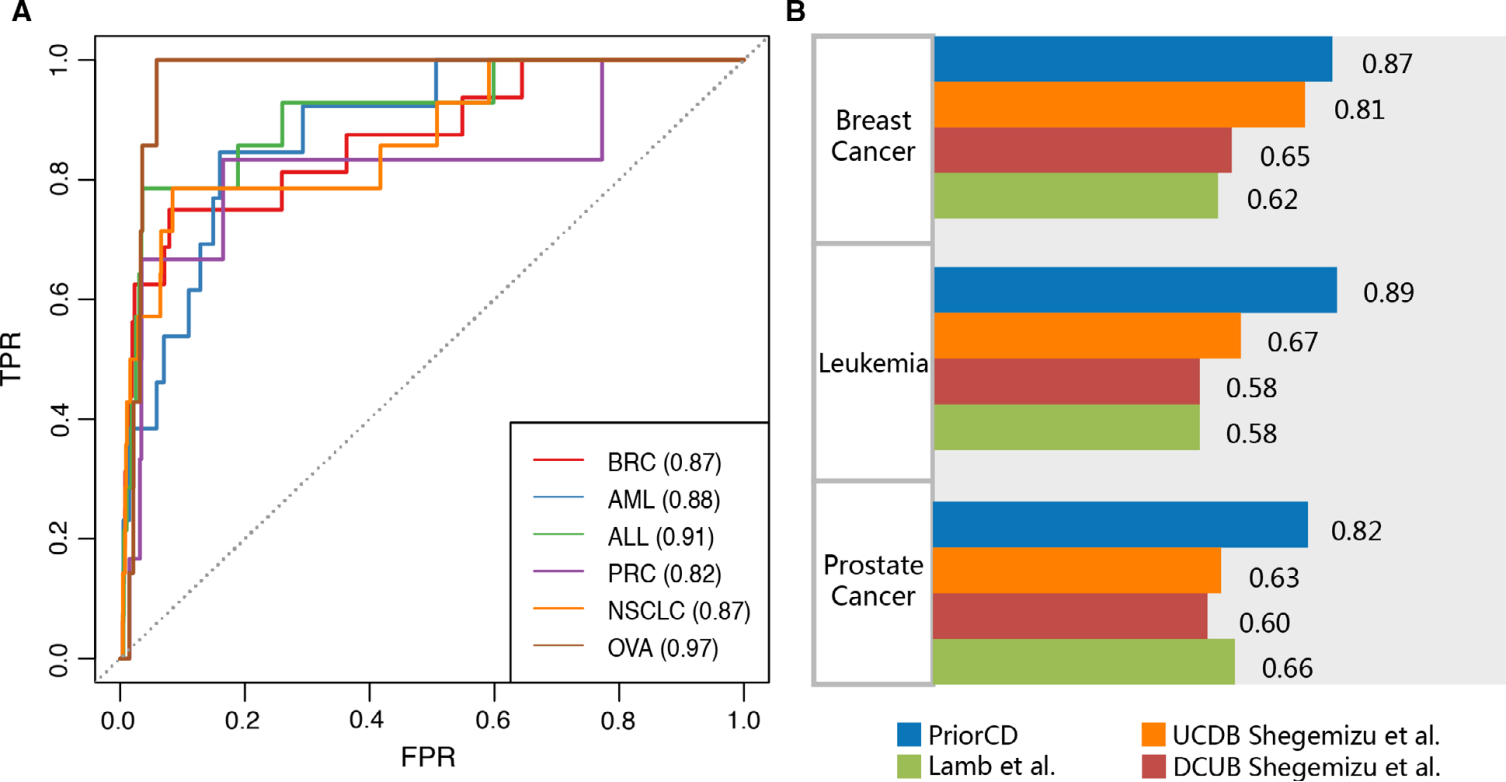
Cross‐validation and comparison results. (A) ROC curves for 6 different cancer drug sets were generated. The AUROC values for each cancer drug set were calculated and were displayed in the brackets, respectively. (B) Comparison between PriorCD and two other methods. We applied PriorCD on three different drug datasets to compare its performance with Lamb *et al*. and Shigemizu *et al*. The TPR and FPR were calculated, and then, AUC values behind the color bar were used to measure their performance. UCDB: drugs that can down‐regulate up‐regulated cancer genes. DCUB: drugs that can up‐regulate down‐regulated cancer genes.

For a more comprehensive confirmation of the accuracy and wide applicability of PriorCD, we also collected therapeutic drug information for four other cancers, acute myeloid leukemia (AML), acute lymphoblastic leukemia (ALL), prostate cancer (PRC), and non‐small‐cell lung cancer (NSCLC), which are shown in Table S2. We considered the restart set (FDA‐approved drugs) of all six types of cancers as the true‐positive drug set. LOOCV and ROC analysis were then used to evaluate the predictability of our PriorCD method. As shown in Fig. 5A, the value of AUROC of our method applied to the breast cancer data set (BRC) was 0.87 and to the ovarian cancer data set (OVA) was 0.97. For the other four types of cancers (AML, ALL, PRC, and NSCLC), it was 0.88, 0.91, 0.82, and 0.87, respectively. The full set of drugs for these types of cancers is shown in [Link], [Link], [Link], [Link].

### 3.5 Comparison of PriorCD with other methods

We also compared the predictability our PriorCD method with two classical computational methods for drug repurposing, proposed by Shigemizu *et al*. (2012) and CMap by Lamb *et al*. (2006), both of which are mainly based on the reverse correlation between gene expression and disease signature. We obtained 1251, 1079, and 1182 compound activity profiles from CMap in breast cancer, leukemia and prostate cancer cell lines, respectively. Gene expression data in breast cancer, leukemia, and prostate cancer cells (GSE6883, GSE5788, and GSE3325) were downloaded from Gene Expression Omnibus (GEO) database of the National Center of Biotechnology Information (NCBI). Subsequently, these two methods were used to generate ranked drug lists. In order to perform ROC curve analysis to evaluate the predictability of the two previous methods, 20 breast cancer drugs, 10 myeloid leukemia (ML) drugs, and 11 prostate drugs that receiving FDA marketing approval were collected and been considered as true‐positive sets. According to the AUROC values, PriorCD shows better values than the two methods above (Fig. 5B).

### 3.6 Robustness analysis of PriorCD method

The KEGG database contains distinct pathways that consist mostly of the same genes, which might lead to highly similar pathway activities across all cancer cell lines. We examined if overlapping pathways influenced our results. Thus, we excluded the highly overlapping pathways and then re‐implemented our method. Specifically, according to the method of eliminating redundancy proposed by Haider *et al*. (2018), we tested the overlap between each pair of pathways and eliminated the smaller one when a pair of pathways had a two‐way overlap above 80% (if two pathways shared over 80% of their genes). In the end, we obtained 199 mRNA pathways and 102 microRNA pathways, which we called nonredundant KEGG (nonreKEGG) pathways. Then, cell–cell and tissue‐of‐origin correlations based on nonrecKEGG pathways were calculated, and the results are shown in Fig. S4. For example, for mRNA pathway activity correlation from different cell lines, the average correlation was 0.959, with a range of 0.863–0.994 and a stand deviation of 0.019. Within a single tissue, the average mRNA pathway activity correlation was 0.977, with a range of 0.963–0.986 and a stand deviation of 0.008. For that between different tissues, the average correlation decreased to 0.958, with a range of 0.925–0.976 and a stand deviation of 0.014. These results were fairly consistent with the previous results derived from original KEGG pathways.

Furthermore, in order to test whether the redundant pathways result in bias results, we then compared the number of overlapping drugs on the top 30 of prioritized drug lists based on KEGG pathways and nonreKEGG pathways. We found that the overlapping ratios of drugs in breast cancer, leukemia, and prostate cancer were 76.7%, 50%, and 73.3% (Fig. S5). These results indicate that the redundant pathways slightly influence the results, and our method is robust and stable for the redundant pathways.

Besides, in order to evaluate whether our method is robust and stable when using different pathway database, mRNA and microRNA were, respectively, enriched into 642 mRNA pathways and microRNA pathways based on Reactome database. Cell–cell and tissue‐of‐origin correlations based on Reactome pathways are shown in Fig. S6. For mRNA pathway activity correlation from different cell lines, the average correlation was 0.976, with a range of 0.921–0.996 and a stand deviation of 0.011. Within a single tissue, the average mRNA pathway activity correlation was 0.986, with a range of 0.978–0.991 and a stand deviation of 0.005. For that between different tissues, the average correlation was 0.974, with a range of 0.954–0.984 and a stand deviation of 0.008. We could observe that the results of correlation based on Reactome pathways (Fig. S6) are similar with those based on KEGG pathways.

We then compared overlapping drugs on the top 30 of prioritized drug lists based on both databases of pathway annotations. Figure S7 shows three Venn diagrams representing three different cancers (breast cancer, leukemia, and prostate cancer). The overlapping ratios of drugs in these three cancers almost exceeded 50%, which demonstrates the robustness and stability of our method for the different pathway database.

## 4 Discussion

Drug repurposing is a strategy for identifying new indications for marketed or investigational drugs and can revitalize compounds that have failed in late clinical trial phase or during preclinical research. As we know, a cancer has its unique genetic characteristics even if they come from the same tissue. This is also the reason why patients with same cancer response differently to identical drugs. Repurposing on cancer drugs could offer patients multiple different choices in order to overcome drug resistance and therefore make the treatment more effective. Moreover, by drug repurposing it is also capable to reveal novel targets or pathways that might provide new thoughts in carcinogenesis and cancer treatment. It has become a focus of experts’ attention in drug development, and many methods have been published in this sphere. The most commonly used computational approaches are based on the comparison of the unique characteristics (such as transcriptomics, proteomics, or chemical structures) of a drug against those of another drug and disease (Hieronymus *et al*., 2006; Keiser *et al*., 2009). Biological pathways may help experts further study on the potential function of drugs (Ye *et al*., 2012). In our PriorCD method, we enriched pathway activity profiles based on mRNA and microRNA expression of 60 cancer cell lines from the NCI‐60 panel. We integrated pathway activities with drug activities to construct a drug functional similarity network for prioritizing candidate drugs, and the resulting tool may provide new insight for drug repurposing.

Both mRNA and microRNA expression profiles were taken into account when we constructed the drug functional similarity network, based on sufficient evidence that mRNA and microRNA are involved in surprisingly diverse biological processes associated with cell homeostasis, such as DNA replication, cell cycle, and cell apoptosis (Kwak *et al*., 2010; Mo, 2012). Using pathway activity profiles could observe various changes at a higher biological level, since in that complex cellular biological processes are not just governed by individual genes, but are impacted by many other genes and molecules. Therefore, the use of gene‐level expression data is not comprehensive to understand the biological significance of cell processes.

In comparison with our PriorCD approach, CMap and the method proposed by Shigemizu *et al*. focused on the gene expression profiles in disease versus normal state. Such methods have been influenced and restricted by expression data. Even if disease profiles are reproduced under the similar conditions, different results may be obtained from one instance to the next. Moreover, our drug activity profiles currently cover a wider range of compounds (over 3000).

In our method, we first enriched mRNA and microRNA into mRNA pathways and microRNA pathways and obtained their activity profiles. Then, we correlated the pathway activities to the drug activities across 60 cancer cell lines. After obtaining mRNA pathway–drug correlations and microRNA pathway–drug correlations, we calculated correlation between each pair of drugs across all pathways to construct the drug functional similarity network based on mRNA pathways and microRNA pathways and then integrated them. This process could be easily explained as ‘Correlations of correlations’ approach. Although some pathways behave the same activity pattern across cell lines (Fig. 3), their correlations to all drugs are not identical (Fig. S8). This indicates that these pathways are informative for the drug clustering, although their effects are modest. Specially, there are several pathways that actually differ between the cell lines. For example, ECM‐receptor interaction pathway and histidine metabolism pathway have lower activity in leukemia cell lines and higher activity in lung cancer cell lines (Fig. S1). These pathways are likely the processes that in the end drive the clustering of drugs.

Two drugs were considered functionally similar if they had similar pathway–drug correlations. We chose gefitinib (NSC715055) and afatinib (NSC750691) as concrete examples to interpret the drug similarity network in biological pathway level. Gefitinib and afatinib are both EGFR inhibitors, which target several related pathways, such as EGFR tyrosine kinase inhibitor resistance pathway and ERBB signaling pathway. Figure S9 shows that these two drugs (gefitinib and afatinib) are both positive correlated with the two pathways (EGFR tyrosine kinase inhibitor resistance pathway and ERBB signaling pathway). Moreover, we could observe that correlations between gefitinib and 227 mRNA pathways and those between afatinib and 227 mRNA pathways were generally consistent. And the correlation between gefitinib and afatinib was 0.71 (FDR = 2.43e‐36). The high correlation between them is driven by the consistency of their pathway–drug correlations across all pathways. Therefore, the two drugs were functionally similar and were connected in our drug functional similarity network. The implication is that the drug functional similarity network could reflect the similarities among drugs on a biological functional level, which means the closer the connection between two drugs in this network; the more similar they are functionally. Our results exactly confirm this conclusion. For instance, in our network, drugs for the treatment of breast cancer such as epirubicin (NSC256942) and doxorubicin (NSC123127) were scattered nearby its candidate drug daunorubicin (NSC82151). All of these drugs are topoisomerase II inhibitors (Table S9) that produce anticancer activity by blocking DNA replication and thereby interfering protein synthesis. Since PriorCD uses pathway activities rather than just gene expression data, it is more conducive to find candidate drugs that have similar pharmacological and pharmacodynamic effects.

In this study, we mainly used FDA‐approved drug sets against specific cancers to prioritize candidate drugs. For a given cancer, the PriorCD method could identify drugs that may have different targets but exert the similar functions with the FDA‐approved drugs. Thus, our method may provide alternatives for patients when they are drug resistance for the present drugs. Moreover, our method could find new uses for approved drugs (drug repurposing). In breast cancer dataset, PriorCD found 14 candidate drugs, some of which approved to treat other diseases have been identified as candidates for treating breast cancer in our study. For instance, two approved drugs for non‐small‐cell lung cancer, gefitinib (prioritized score = 9.73E‐03, FDR < 0.001) and afatinib (prioritized score = 3.14E‐03, FDR < 0.001), were ranked at the top of the prioritized list and were considered as candidate drugs for breast cancer. However, there are still a number of drugs that have not been approved by the FDA, which may result in the incomplete restart sets and candidate drugs. As the number of drugs receiving marketing approval increases, so does the completeness of the prioritized list and the robustness and accuracy of our method.

In order to make PriorCD be more widely used, we made an attempt to develop it into a flexible R‐based package ‘PriorCD’, which can be downloaded freely from CRAN (https://cran.r-project.org/web/packages/PriorCD). The drug functional similarity network we constructed in this work can also be obtained from this R‐based package. If users enter a series of therapeutic drugs against a cancer of interests, a prioritized drug list with detailed information will be returned.

## 5 Conclusions

In this study, we proposed a powerful computational method, PriorCD, to prioritize candidate cancer drugs by integrating pathway activity profiles and drug activity profiles. This provides a new approach to drug repurposing by considering the drug functional similarities at the pathway level. The performance of PriorCD was evaluated by using drug datasets of breast cancer and ovarian cancer. PriorCD provided predictions of 14 and 8 candidate drugs for breast cancer and ovarian cancer drug sets. According to cross‐validation test and literature searches, we validated that PriorCD can efficiently prioritize candidate cancer drugs.

## Conflict of interest

The authors declare no conflict of interest.

## Author contributions

JWH and LC conceived and designed the study. JYD and BTZ developed software. QK, YJ, and SYL analyzed the data and implemented the methodology. YPZ and XDH revised the manuscript. YY and YQS provided constructive discussions. JWH and JYD drafted the manuscript. All the authors read and agreed to the manuscript.

## Supporting information


**Fig. S1.** A hierarchical clustering heatmap of 15 cancer cell lines and 227 pathways.
**Fig. S2.** Cell‐cell and tissue‐of‐origin correlation.
**Fig. S3.** A subnetwork of the drug functional similarity of breast cancer.
**Fig. S4.** Cell‐cell and tissue‐of‐origin correlation.
**Fig. S5.** Overlap among top 30 candidate drugs when non‐redundant KEGG (nonreKEGG) pathway annotations and KEGG pathway annotations are used.
**Fig. S6.** Cell‐cell and tissue‐of‐origin correlation.
**Fig. S7.** Overlap among top 30 candidate drugs when Reactome pathway annotations and KEGG pathway annotations are used.
**Fig. S8.** A hierarchical clustering heatmap of correlations of 227 mRNA pathways and 3,652 drugs, where red indicates positive correlations and blue for negative correlations.
**Fig. S9.** Correlations between gefitinib (NSC715055) and 227 KEGG pathways and those between afatinib (NSC750691) and 227 KEGG pathways.

**Table S1.** Drug‐Drug functional similarity network.

**Table S2.** Restart drug set of breast cancer, acute myeloid leukemia, acute lymphoblastic leukemia, prostate cancer, non‐small cell lung cancer, and ovarian cancer.

**Table S3.** Full list of candidate drugs for breast cancer.

**Table S4.** Full list of candidate drugs for ovarian cancer.

**Table S5.** Full list of candidate drugs for acute myeloid leukemia.

**Table S6.** Full list of candidate drugs for acute lymphoblastic leukemia.

**Table S7.** Full list of candidate drugs for prostate cancer.

**Table S8.** Full list of candidate drugs for non‐small cell lung cancer

**Table S9.** Mechanism of action of drug.
