# Supplementary material for: Prioritization of candidate cancer drugs based on a drug functional similarity network constructed by integrating pathway activities and drug activities
Source: Mol Oncol. 2019 Aug 21;13(10):2259–77. doi: 10.1002/1878-0261.12564 (PMC6763777; doi:10.1002/1878-0261.12564)
Supplement: Supplementary file 1 — Fig. S1. A hierarchical clustering heatmap of 15 cancer cell lines and 227 pathways. Fig. S2. Cell‐cell and tissue‐of‐origin correlation. Fig. S3. A subnetwork of the drug functional similarity of breast cancer. Fig. S4. Cell‐cell and tissue‐of‐origin correlation. Fig. S5. Overlap among top 30 candidate drugs when non‐redundant KEGG (nonreKEGG) pathway annotations and KEGG pathway annotations are used. Fig. S6. Cell‐cell and tissue‐of‐origin correlation. Fig. S7. Overlap among top 30 candidate drugs when Reactome pathway annotations and KEGG pathway annotations are used. Fig. S8. A hierarchical clustering heatmap of correlations of 227 mRNA pathways and 3,652 drugs, where red indicates positive correlations and blue for negative correlations. Fig. S9. Correlations between gefitinib (NSC715055) and 227 KEGG pathways and those between afatinib (NSC750691) and 227 KEGG pathways. [file MOL2-13-2259-s001.docx]

**
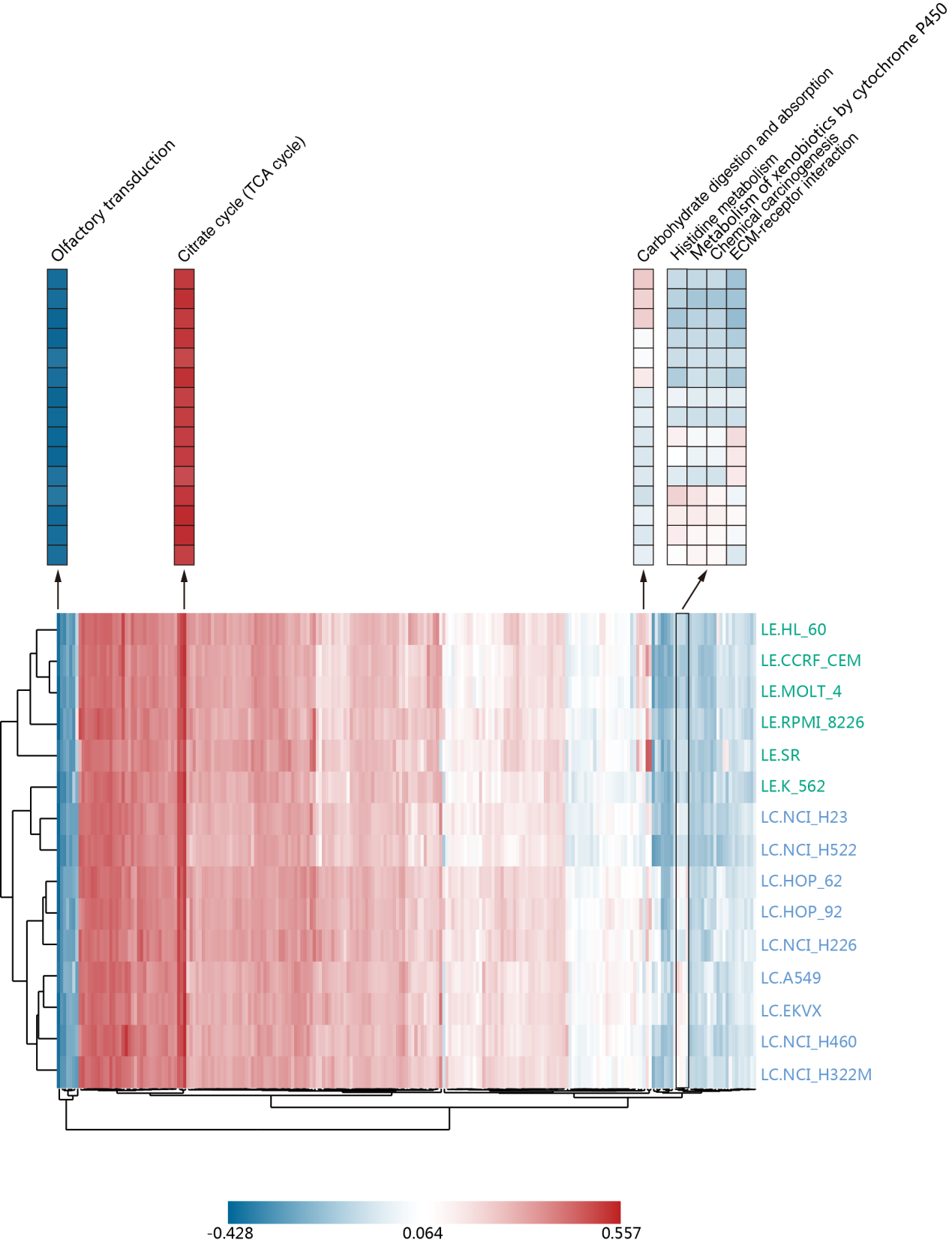
**

**Supplementary Figure S1.** A hierarchical clustering heatmap of 15 cancer cell lines and 227 pathways. A great majority of pathways have almost the same activity pattern, such as the activity of citrate cycle (TCA cycle) pathway and olfactory transduction pathway. Only a few pathways acted distinctly different in the two tissues, such as ECM-receptor interaction pathway, histidine metabolism pathway, and Carbohydrate digestion and absorption pathway.


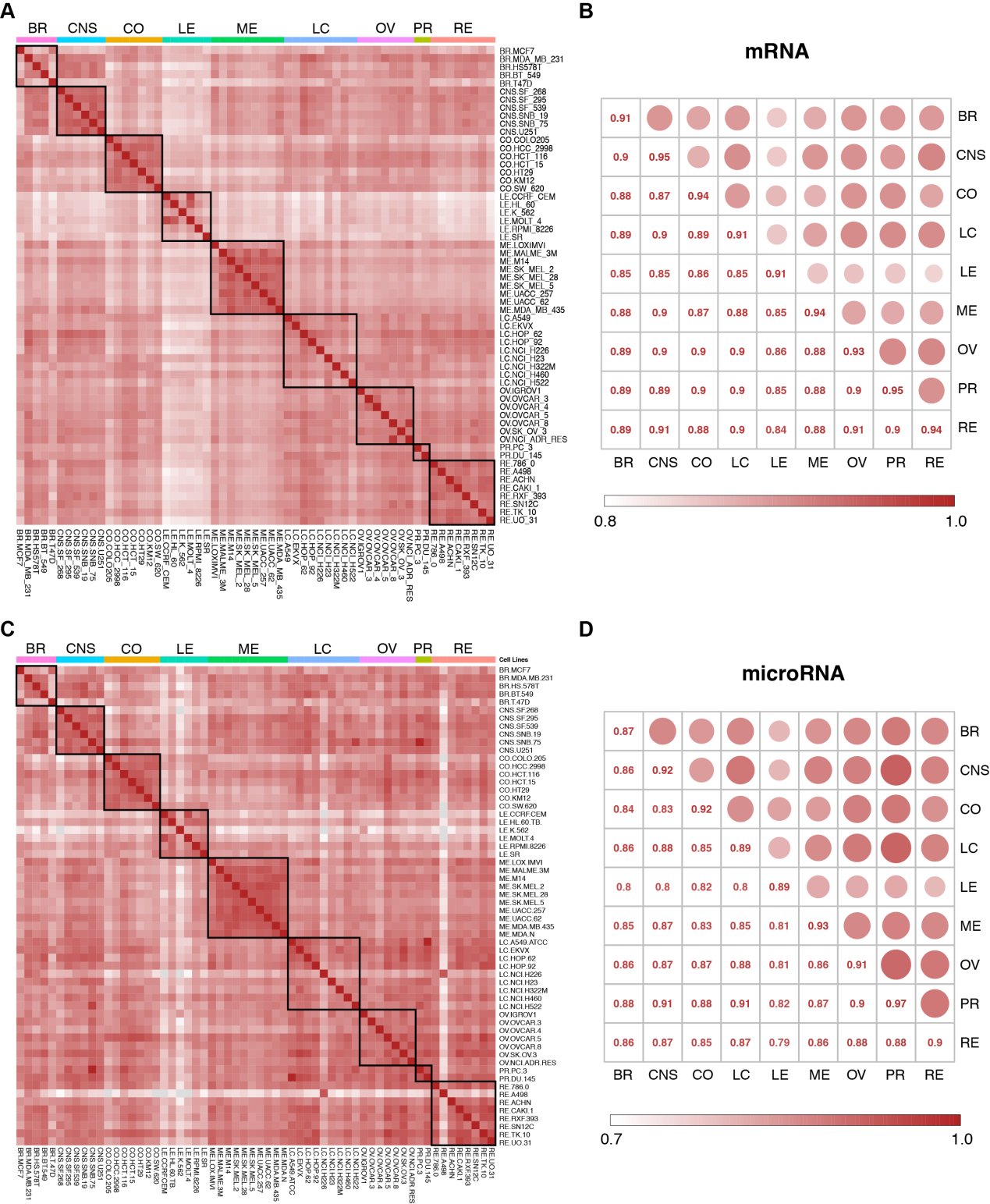


**Supplementary Figure S2.** Cell-cell and tissue-of-origin correlation. Pearson correlation coefficient (PCC) of 19,794 mRNAs and 319 microRNAs expression profiles respectively, presented at the levels of NCI-60 cell line and tissue of origin. (A) Heatmap of cell-cell correlation coefficient for mRNA. (B) Mean tissue of origin correlation coefficient for mRNA. (C) Heatmap of cell-cell correlation coefficient for microRNA. (D) Mean tissue of origin correlation coefficient for microRNA.

**
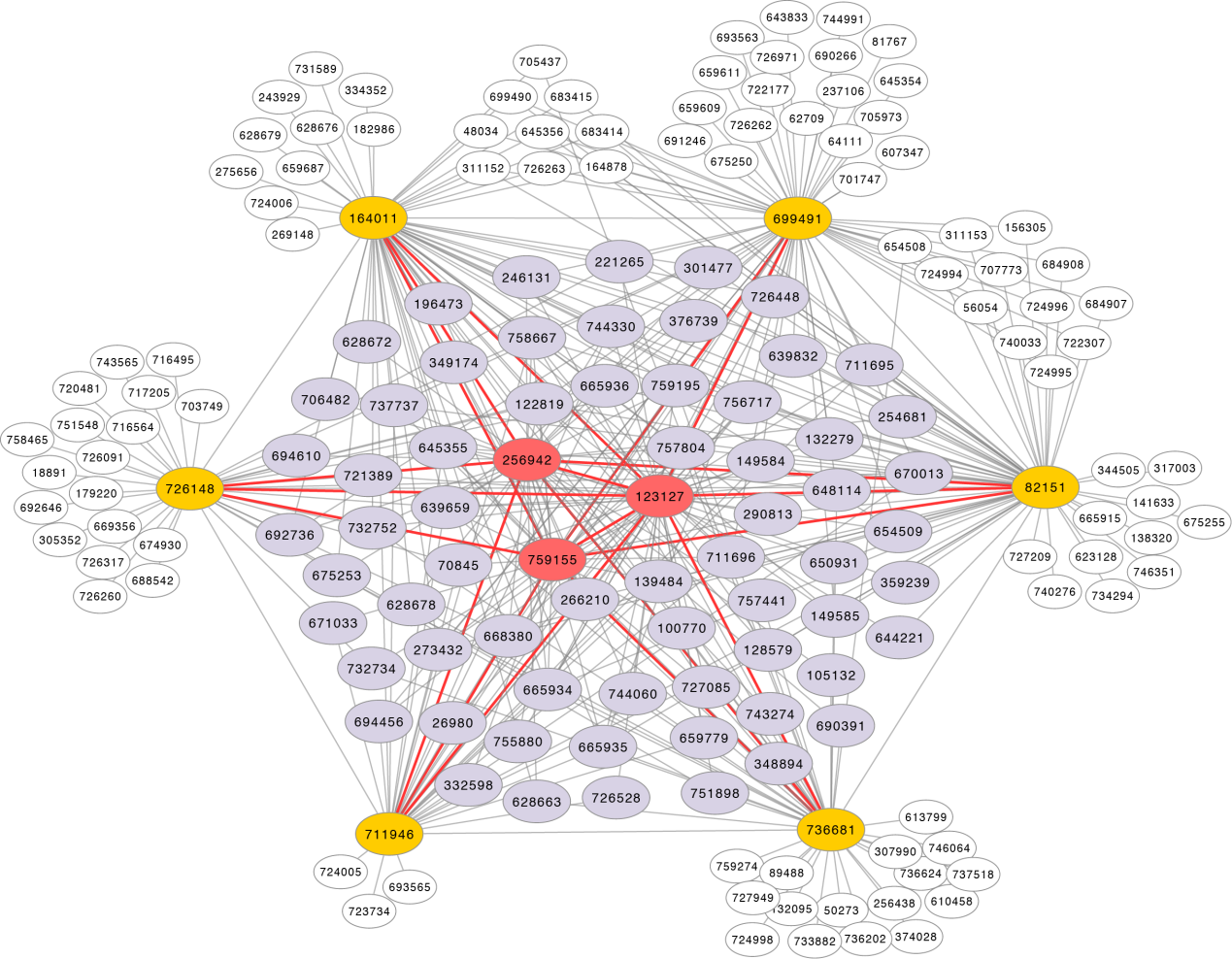
**

**Supplementary Figure S3.** A subnetwork of the drug functional similarity of breast cancer. The subnetwork of the drug functional similarity network associated with breast cancer comprised three restart drugs in red (NSC123127, NSC256942 and NSC759155) and five candidate drugs (NSC82151, NSC164011, NSC699491, NSC711946, NSC726148 and NSC736681) in yellow. Grey nodes represent shared neighbours of restart drugs and candidate drugs. The RWR algorithm scores these candidate drugs higher because they have more direct neighbours and shared neighbours in restart drug set.


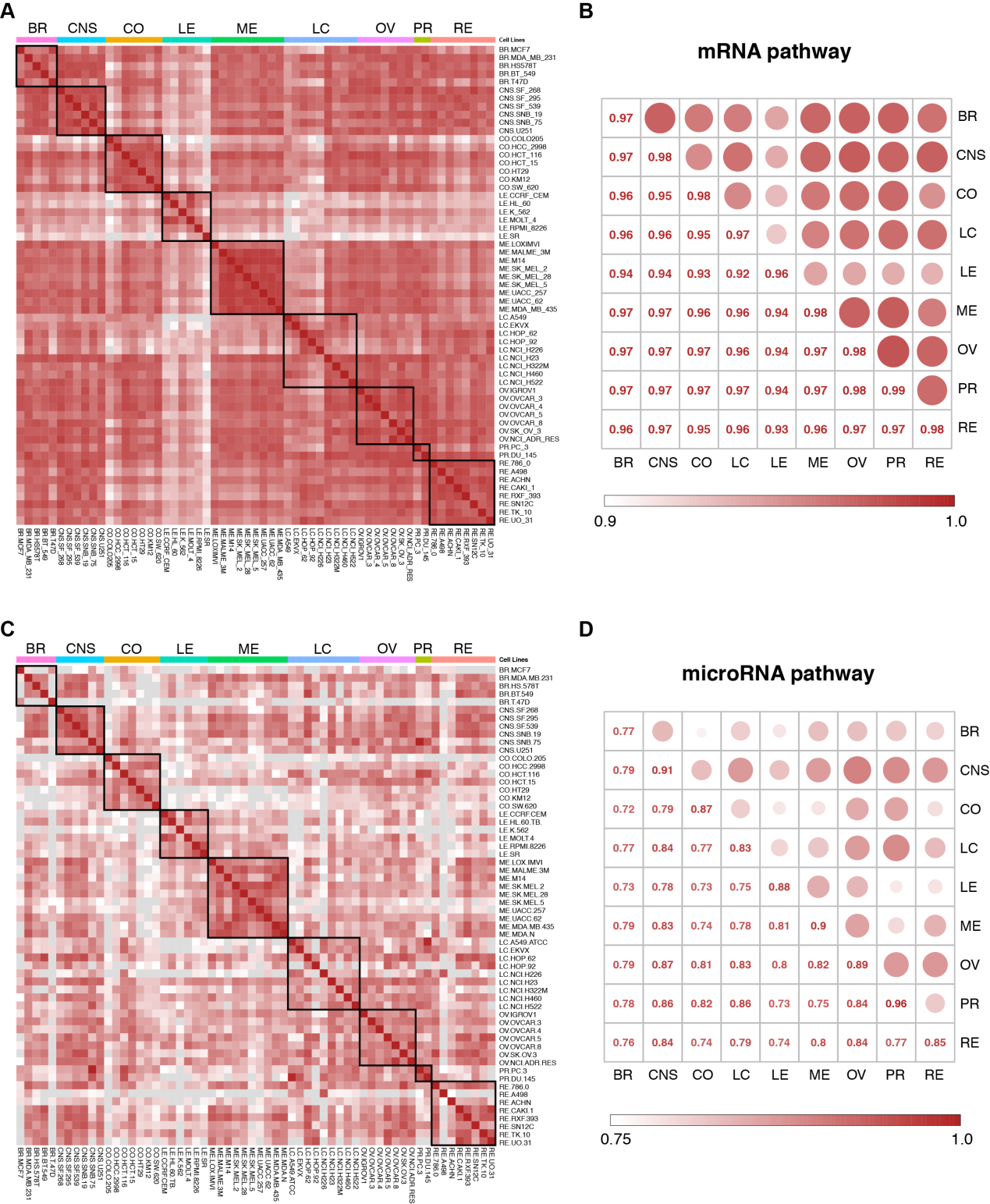


**Supplementary Figure S4.** Cell-cell and tissue-of-origin correlation. Pearson correlation coefficient (PCC) of 199 mRNA and 102 microRNA non-redundant KEGG (nonreKEGG) pathway activity profiles respectively, presented at the levels of NCI-60 cell line and tissue of origin. (A) Heatmap of cell-cell correlation coefficient for mRNA nonreKEGG pathway. (B) Mean tissue of origin correlation coefficient for mRNA nonreKEGG pathway. (C) Heatmap of cell-cell correlation coefficient for microRNA nonreKEGG pathway. (D) Mean tissue of origin correlation coefficient for microRNA nonreKEGG pathway.


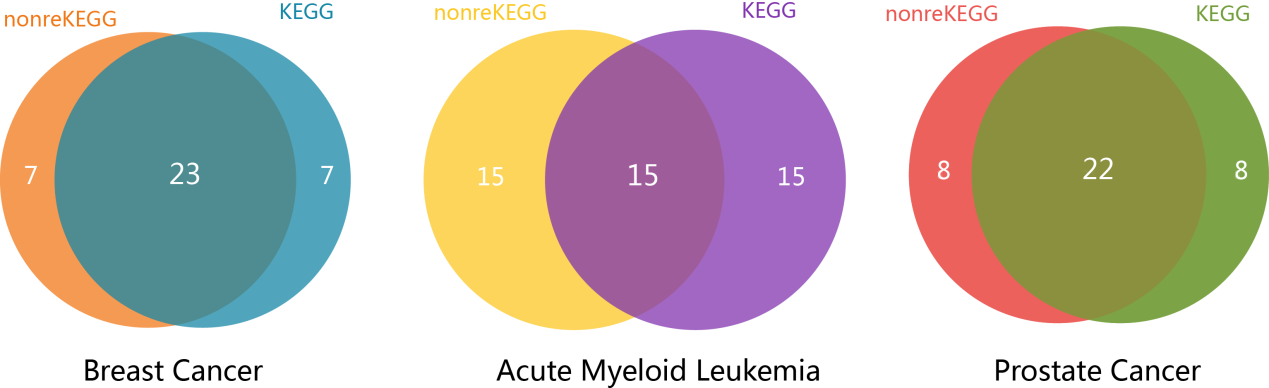


**Supplementary Figure S5.** Overlap among top 30 candidate drugs when non-redundant KEGG (nonreKEGG) pathway annotations and KEGG pathway annotations are used.


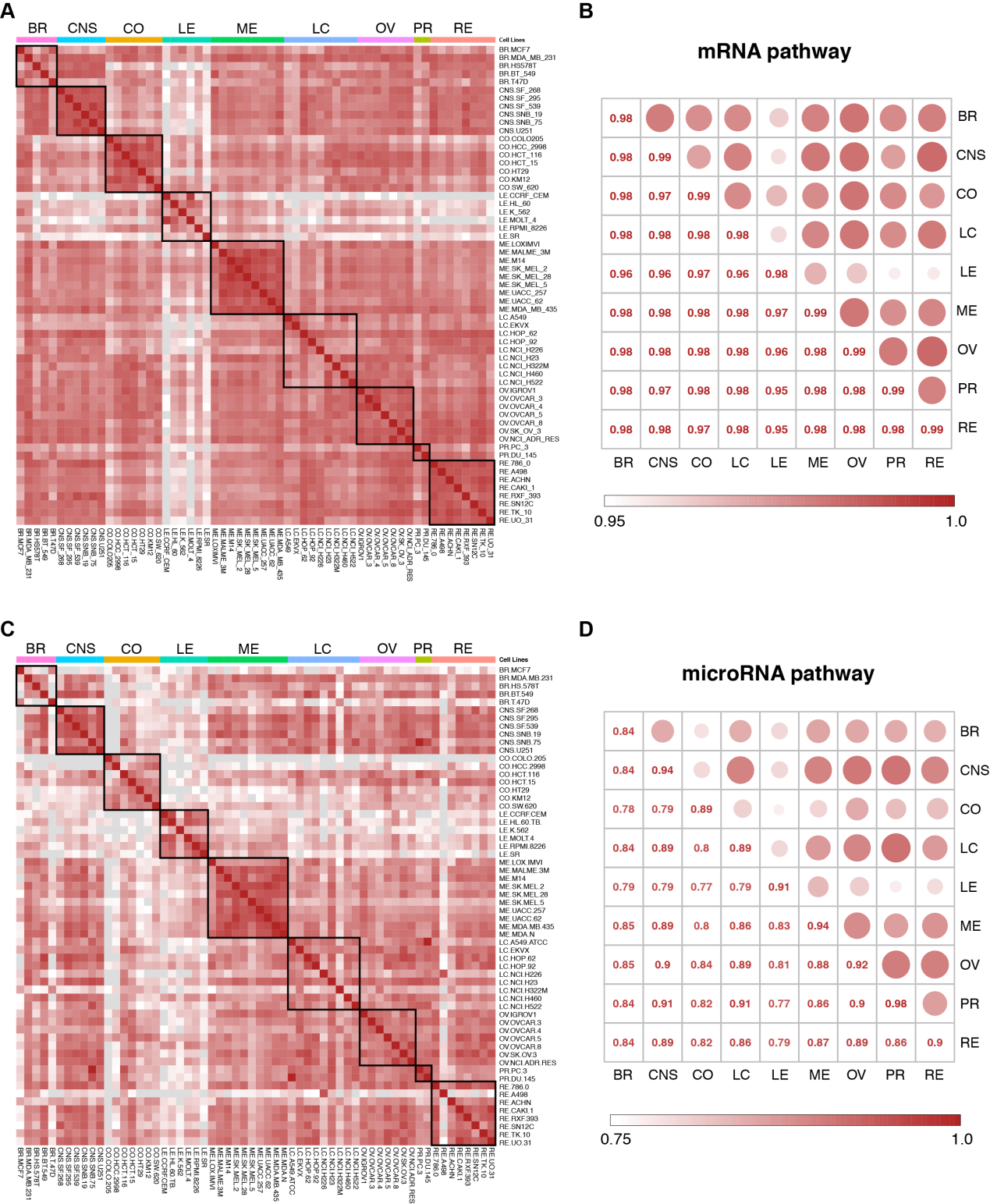


**Supplementary Figure S6.** Cell-cell and tissue-of-origin correlation. Pearson correlation coefficient (PCC) of 642 mRNA and 315 microRNA Reactome pathway activity profiles respectively, presented at the levels of NCI-60 cell line and tissue of origin. (A) Heatmap of cell-cell correlation coefficient for mRNA Reactome pathway. (B) Mean tissue of origin correlation coefficient for mRNA Reactome pathway. (C) Heatmap of cell-cell correlation coefficient for microRNA Reactome pathway. (D) Mean tissue of origin correlation coefficient for microRNA Reactome pathway.


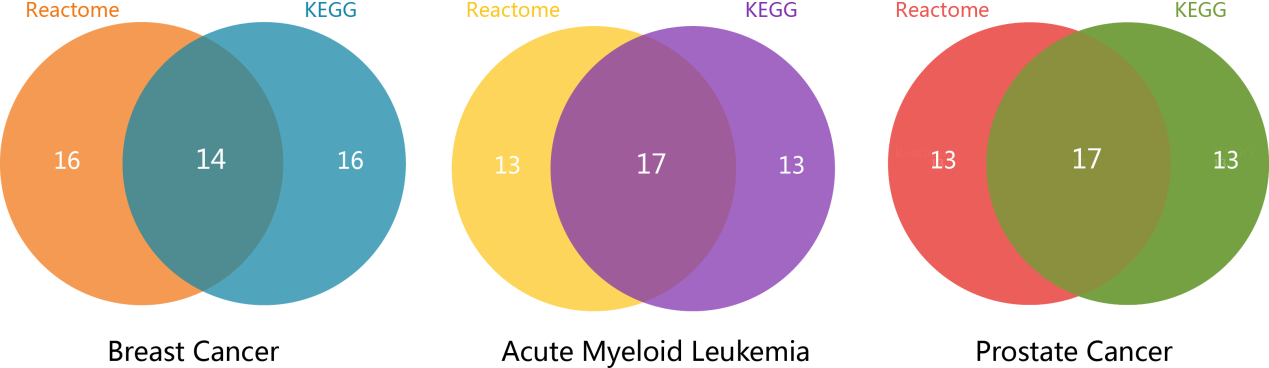


**Supplementary Figure S7.** Overlap among top 30 candidate drugs when Reactome pathway annotations and KEGG pathway annotations are used.


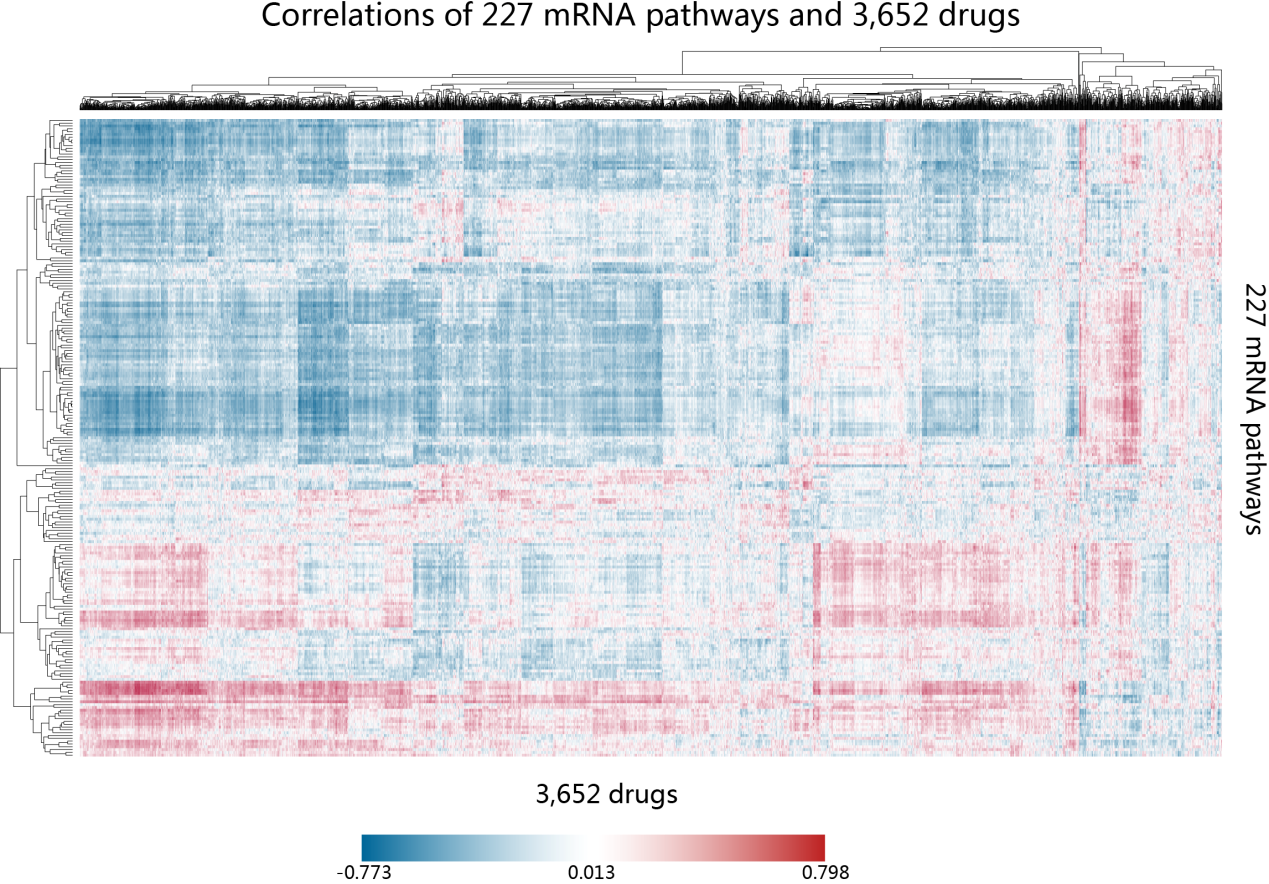
**Supplementary Figure S8.** A hierarchical clustering heatmap of correlations of 227 mRNA pathways and 3,652 drugs, where red indicates positive correlations and blue for negative correlations.


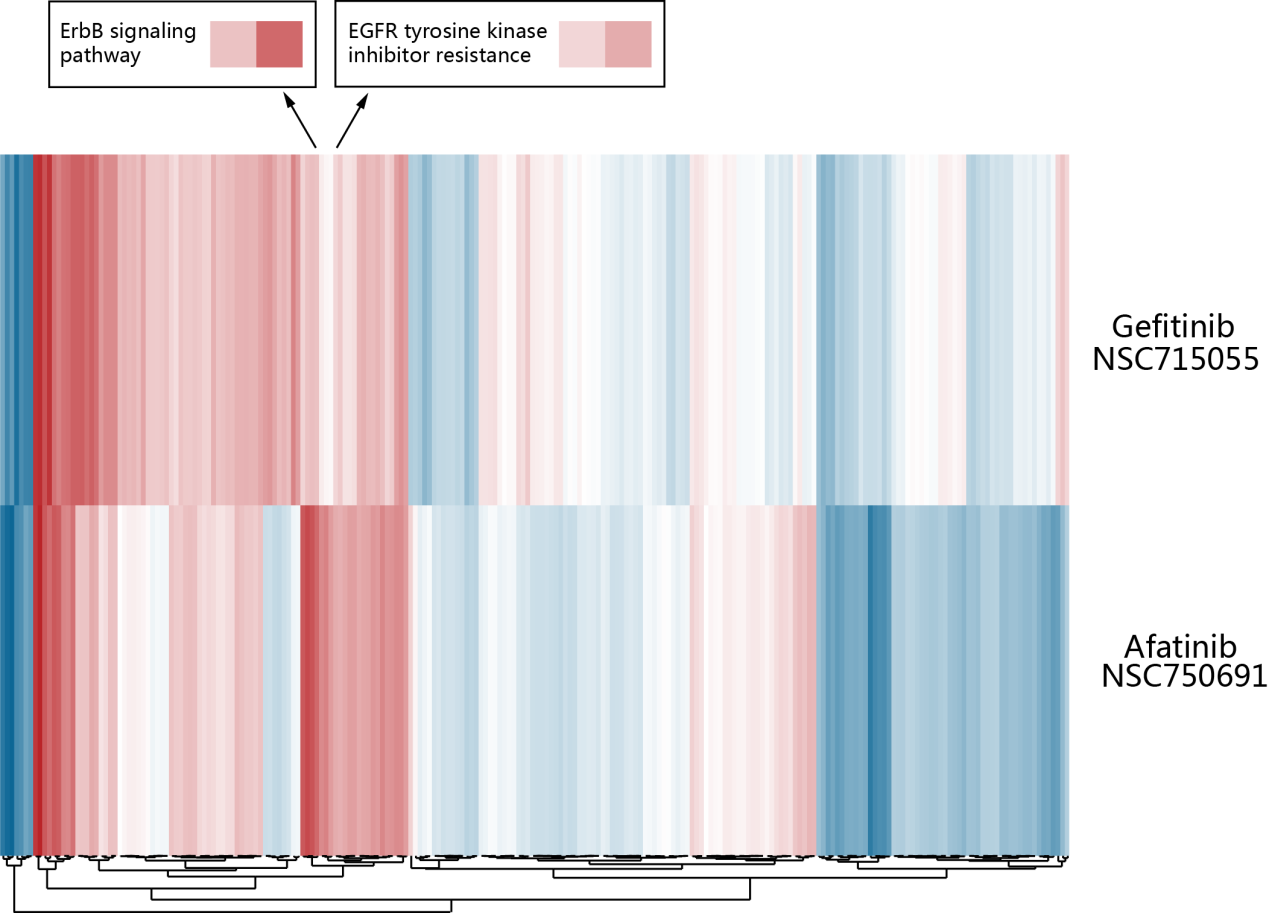


**Supplementary Figure S9.** Correlations between gefitinib (NSC715055) and 227 KEGG pathways and those between afatinib (NSC750691) and 227 KEGG pathways. Gefitinib and Afatinib have similar activity pattern. And they show positive correlations to their targeting pathways, such as EGFR tyrosine kinase inhibitor resistance pathway and ErbB signaling pathway.
